# Atherosclerotic and Cardio-Metabolic Diseases: From Molecular Basis to Therapeutic Advances

**DOI:** 10.3390/ijms24119737

**Published:** 2023-06-04

**Authors:** Eva Kassi, Ioannis Kyrou, Harpal S. Randeva

**Affiliations:** 1 Department of Biological Chemistry, Medical School, National and Kapodistrian University of Athens, 11527 Athens, Greece; 2 Endocrine Unit, 1ST Department of Propaedeutic Internal Medicine, Laiko Hospital, National and Kapodistrian University of Athens, 11527 Athens, Greece; 3 Warwick Medical School, University of Warwick, Coventry CV4 7AL, UK; kyrouj@gmail.com; 4 Warwickshire Institute for the Study of Diabetes, Endocrinology and Metabolism (WISDEM), University Hospitals Coventry and Warwickshire NHS Trust, Coventry CV2 2DX, UK; 5 Research Institute for Health & Wellbeing, Coventry University, Coventry CV1 5FB, UK; 6 Aston Medical School, College of Health and Life Sciences, Aston University, Birmingham B4 7ET, UK; 7 Laboratory of Dietetics and Quality of Life, Department of Food Science and Human Nutrition, School of Food and Nutritional Sciences, Agricultural University of Athens, 11855 Athens, Greece

Cardiovascular diseases (CVDs) still remain the major cause of death worldwide; however, CVD-related mortality has been reduced due to lifestyle modification interventions, as well as novel pharmacological therapies and advances in cardiovascular surgery. Notably, a spectrum of diseases which are closely connected with atherosclerosis, such as non-alcoholic fatty liver disease (NAFLD), dyslipidaemia, arterial hypertension, and metabolic syndrome, appear to share common molecular pathogenetic mechanisms. In the present Special Issue, we focus on this spectrum of atherosclerosis-related cardio-metabolic diseases, aiming to offer a better understanding of their common molecular backgrounds, which can further help in designing prognostic/diagnostic tools and developing novel therapeutic tools.

In their review, Meng et al. describe and discuss the latest methods in the early diagnosis of coronary atherosclerosis via imaging, the evaluation of gene and protein markers, and trace elements [1]. Among imaging techniques, computed tomography coronary angiography (CCTA) can replace invasive coronary angiography (ICA) in individuals with suspected acute coronary syndrome (ACS) who have a low or medium pre-test risk of coronary artery disease (CAD). Furthermore, multiple studies have reported numerous miRNAs that are expressed in ACS and stable CAD, with miR-1, miR-133, miR-208a, and miR-499 considered to be ACS biomarkers. However, the authors noted that the use of such miRNAs in clinical practice needs additional work to address any current methodological, technical, or analytical shortcomings.

In the review by Kowara and Cudnoch-Jedrzejewska, different approaches directed at the specific molecular pathways involved in atherosclerotic plaque vulnerability and destabilisation are presented [2]. Metabolic approaches target lowering LDL particles and increasing HDL, whilst cell survival approaches aim at promoting cell survival mechanisms, such as efferocytosis and autophagy in VSMC and macrophages, as well as the M2 anti-inflammatory phenotype polarisation of macrophages. Furthermore, anti-inflammatory approaches focus on a reduction in T cell infiltration and migration as well as cytotoxic T cell activity, while antioxidant approaches are directed towards the neutralisation of reactive oxygen species. Finally, targeting intra-plaque neovascularisation via the inhibition of VEGF receptors and extracellular matrix (ECM) fragmentation is considered to be plaque-stabilising. As the authors discuss, microRNAs emerge as a potential therapeutic option, as they can control all stages of atherosclerotic plaque progression via regulating the corresponding mRNAs. Thus, applying agomirs of stabilising miRNAs or antagomirs of destabilising miRNAs could be promising in the prevention of plaque vulnerability.

Interestingly, according to the “timing hypothesis”, HRT appears to be safe and even protective in younger postmenopausal women who are asymptomatic for CVD and within 10 years of menopause onset [3]. This means that, once atherosclerotic plaque has been formed, oestrogen may be potentially harmful. The favourable effects of oestrogen in the early stages of the atheromatosis process (endothelium activation/dysregulation) have been widely investigated; however, data on the role of oestrogen in the factors involved in the later stages of the atherogenesis process which lead to plaque vulnerability/rupture are insufficient.

Nasiri-Ansari et al. aimed to investigate the effects of oestradiol (E_2_) on the expression of the molecules implicated in atherosclerotic plaque vulnerability, resembling a low-grade inflammatory state that occurs in atherosclerosis, using an established endothelial in vitro system [4]. They also tried to clarify whether these effects are mediated by ERα, ERβ, or GPR-30 receptors. Their research findings showed that in the absence of low-grade inflammation, an overexpression of ERα can initiate the atherosclerosis process via increasing the expression of MCP-1 and MMP-2 activity. Moreover, under low-grade inflammation, E_2_ can promote the destabilisation of atherosclerotic plaque via increasing the expression of MCP-1 and MMP-9, as well as the activity of MMP-2 in human arterial endothelial cells (HAECs), an effect mediated via GPR-30. Accordingly, the authors conclude that the balance of the expression of the various ER subtypes may play an important role in the paradoxical characterisation of oestrogen as being both beneficial and harmful, since E_2_ induces different effects in either the early stages of atheromatosis or atheromatous plaque instability via different ERs. Further studies will identify the exact role of each ER subtype in the atherosclerosis inflammatory process, which could potentially lead to the development of specific ER agonists/antagonists with an improved benefit/risk ratio.

Research on novel heart failure (HF) biomarkers is currently being strongly promoted, particularly using a multi-marker rather than a single-biomarker approach. Among such biomarkers is the soluble suppression of tumorigenesis-2 (sST2), which acts as a decoy receptor for IL-33. Indeed, sST2 has been shown to be associated with endothelial dysfunction, acute decompensated heart failure, myocardial fibrosis, and adverse remodelling.

Dimitropoulos et al., in their research article, examined the association between arterial wall properties and sST2 levels in patients with HF of ischemic aetiology [5]. Their findings show that sST2 levels were associated with the HF status and functional capacity of the patients as assessed using the NYHA classification, although the levels of sST2 were not associated with left ventricular ejection fraction (LVEF). No association between sST2 and pulse wave velocity (PWV, a marker of arterial stiffness) was found; however, an inverse association between sST2 levels and FMD was observed, underscoring the interplay between endothelial dysfunction and HF pathophysiologic mechanisms. Although the authors note that a conclusion about a causal connection between sST2 and endothelial dysfunction in patients with HF of ischemic aetiology cannot be supported by this study, their findings add additional information on endothelial dysfunction occurring in those with decompensated heart failure underlined by myocardial remodelling/fibrosis. Further studies are needed before suggesting sST2 with prognostic significance in clinical practice.

Chen et al. investigated whether treatment with endothelial progenitor cells (EPCs) derived from patients with peripheral arterial occlusive disease (PAOD) would protect limbs against critical limb ischemia (CLI) in adult male nude mice [6]. Notably, they found that only rejuvenated EPCs (the EPCs received combined CD34^+^ cell and hyperbaric oxygen treatment) could restore blood flow and salvage CLI in this mouse model. Although the underlying mechanisms may differ between mice and humans and the exact mechanistic basis of xenogeneic EPC therapy still needs to be fully elucidated, the rejuvenated EPC therapy may serve as an innovative therapy for CLI/severe PAOD patients.

SGLT2 inhibitors (SGLT2is) have been approved for use as anti-diabetic medication due to their favourable effects on reducing hyperglycaemia and metabolism via increasing glucosuria and natriuresis. Additional effects of SGLT2is have been recognised, such as weight loss due to urinary loss of calories, improved renal function and blood pressure, and direct actions in the myocardium. Moreover, SGLT2i treatment has been shown to improve insulin β cell function and insulin sensitivity in patients with type 2 diabetes mellitus (T2DM). In the research article by Taberner-Cortés et al., the possible benefit of dapagliflozin (a selective SGLT2i) in atherosclerosis was investigated using an *ApoE^−/−^ Irs2^+/−^* mouse model. Under atherogenic dietary conditions, it exhibited insulin resistance and accelerated atherosclerosis, but not hypertension or hyperglycaemia [7]. According to their results, dapagliflozin reduced glucose-stimulated insulin secretion from islets and insulin-signalling in adipose tissue, without altering glucose levels. Moreover, in contrast with other studies showing that SGLT2is can attenuate the atherosclerosis process, the authors found that treatment with dapagliflozin did not affect the atherosclerosis lesion size and plaque stability, nor the circulating inflammatory markers [8,9]. Further research will elucidate whether or not the beneficial effects of SGLT2is on the atherosclerosis process are exerted only in the presence of insulin resistance and hypercholesterolemic conditions.

A comprehensive review by Sagris et al. summarised the existing data on the pre-disposing factors of atrial fibrillation (AF), including dietary habits, sedentary lifestyle, and potential genetic factors which regulate pathogenic mechanisms, such as fibrosis, oxidative stress, and inflammatory processes [10]. The latter includes mutations/polymorphisms in potassium and sodium channel genes, as well as mutations in the gap junctional protein. The authors emphasised that lifestyle modifications including alcohol reduction and Mediterranean diet combined with extra virgin olive oil, weight loss, and cardio-metabolic risk factor management are fundamental in AF prevention. Moreover, medications, such as anti-inflammatory agents, angiotensin-converting enzyme inhibitors, angiotensin receptor blockers, aldosterone antagonists, and the novel SGLT2i, have revealed beneficial effects on AF inducibility.

Aguilar-Ballester et al., in their review, present and discuss new pharmacological treatments which, in addition to their anti-diabetic and lipid-lowering effects, appear to also have direct beneficial effects on CVD [11]. As such, incretin-based therapies, SGLT2is, and proprotein convertase subtilisin kexin 9 inactivating therapies (PCSK9is) can act directly on the cells implicated in the atherosclerosis process (endothelial, VMCs, immune cells), as well as on cardiomyocytes. Within this work, the authors summarised experimental studies and randomised control trials (RCTs) which provide strong evidence for the potential effects of these new treatments on the cardiovascular system, beyond metabolic control.

Sestrin2 is an antioxidant protein that was originally identified as hypoxia-induced gene 95 (Hi95). It is expressed and secreted mainly by macrophages, T lymphocytes, endothelial cells, cardiac fibroblasts, and cardiomyocytes [12]. Kishimoto et al. analysed the protective role of sestrin2 against the progression of atherosclerotic and cardiac diseases [13]. From a pathophysiological point of view, sestrin2 appears to be upregulated as a response to stress stimuli (i.e., oxidative, ER, and genotoxic stress) and activates two signalling pathways —Kelch-like ECH-associated protein 1 (Keap1)/Nrf2 and AMPK/mTORC1— which leads to decreased ROS accumulation, apoptosis and inflammation, and increased autophagy. Clinical studies have shown that the plasma levels of sestrin2 were positively correlated with the severity of both CAD and carotid atherosclerosis, possibly reflecting a compensatory response to increased oxidative stress. However, further studies are needed to elucidate the exact role of sestrin2 and its potential therapeutic use in atherosclerotic diseases.

A growing body of evidence suggests that adipose tissue anatomic specificity is pivotal to the pathophysiology of various cardio-metabolic diseases [14]. Specifically, epicardial adipose tissue (EAT) has emerged as a very interesting fat depot, which surrounds the coronary arteries and appears to have specific metabolic properties. Of note, several studies have identified EAT as being an independent predictor of CAD. The review by Conceicão et al. published in this Special Issue provides an overview of the potential effects of dysfunctional EAT on CAD pathophysiology [15]. Using integrative bioinformatics analysis, they identified 46 up- or downregulated proteins which are mainly implicated in inflammatory processes to modulate the local environment and the formation and progression of coronary atherogenesis. Future studies will assess whether targeting the paracrine and endocrine communication between EAT and coronary arteries (e.g., via molecules with anti-inflammatory properties) could be considered as a promising therapeutic approach for those with CAD.

Coronary artery ectasia (CAE) is a relatively common coronary angiographic finding which is associated with atherosclerosis in 50% of cases [16]. In their review article, Richards et al. presented the proposed mechanisms underlying vascular remodelling in the pathogenesis of CAE [17]. Although CAE and CAD share common pathogenetic pathways, such as oxidative stress, vascular endothelial dysfunction, lipid dysregulation, and altered extracellular matrix regulation, there are significant differences in the cytokine milieu and metabolite profile in CAE compared to CAD, which strongly implies that there is a distinct pathogenesis of CAE that potentially requires a different therapeutic approach.

NAFLD refers to a spectrum ranging from benign liver steatosis to non-alcoholic steatohepatitis (NASH), liver fibrosis, and eventually cirrhosis and hepatocellular carcinoma. Over recent decades, NAFLD has evolved as a major health problem. Notably, considering its underlying pathogenesis, which involves mechanisms such as ER stress, apoptosis, and autophagy, as well as the spectrum of NAFLD-associated cardio-metabolic disorders, the term metabolic (dysfunction)-associated fatty liver disease (MAFLD) has been proposed [18,19,20].

Flessa et al. presented the most common mouse models used in NAFLD research [21]. Notably, several such models are being used, involving dietary interventions (e.g., the MCD, AMLN, GAN, and fast-food-like diets), genetic manipulations (e.g., *Prostaglandin E2*-deficient mice and the APOE2ki, *ApoE*^−/−^, *Krt18*^−/−^, *Mat1a*^−/−^, and NEMO^LPC-KO^ mice), the administration of chemical substances (e.g., carbon tetrachloride, CCl_4_; streptozotocin), and/or a combination of these to replicate either the entire NAFLD spectrum or a particular disease stage (e.g., NASH). Given that there is no ideal animal model for the NAFLD spectrum yet, the specific research hypothesis/objectives of each study will drive the selection of the most suitable model in order to investigate the NAFLD-related pathophysiologic mechanisms and/or treatments.

SGLT2is have been investigated as a potential treatment for NAFLD. The exact molecular mechanisms mediating these effects have not been fully elucidated. In their narrative review, Androutsakos et al. provided an overview of the current evidence on the mechanisms underlying the pathogenesis of NAFLD and the potential impact of SGLT2is on NAFLD development and progression, summarising data from in vitro, animal, and human studies [22]. Relevant data indicate that a reduction in hyperglycaemia, improvement in systematic insulin resistance, increased caloric loss, and decreased body weight mostly due to glycosuria contribute to the alleviation of NAFLD. Apart from this, SGLT2is exert a hepato-protective effect by decreasing hepatic inflammation and hepatic de novo lipogenesis and increasing hepatic beta-oxidation. An augmented number of clinical studies have highlighted the favourable effects of SGLT2is on NAFLD in patients with T2DM, estimated using non-invasive biomarkers, imaging techniques, or even liver biopsy, while data on non-diabetic patients remain limited. Interestingly, the authors noted that different effects between members of the SGLT2i class have been observed, suggesting that there are features specific to these individual drugs regarding the underlying mechanism(s) of action and their corresponding effects on NAFLD. Future mechanistic studies would expand our understanding of the specific mechanisms underlying the pathogenesis of NAFLD and the potential favourable actions of SGLT2is for NAFLD treatment.

Nasiri-Ansari et al., in their research article published in this Special Issue, investigated the impact of empagliflozin (a selective SGLT2i) in NAFLD using high fat diet (HFD)-fed ApoE^(−/−)^ mice [23]. Empagliflozin led to reduced fasting glucose, as expected, but also decreased the NAFLD activity score. The latter was accompanied by a decreased expression of lipogenic enzymes (*Fasn*, *Screbp-1c*, and *Pck-1*) and inflammatory molecules (*Mcp-1* and *F4/80*). Focusing on the underlying mechanisms, the authors demonstrated for the first time that empagliflozin treatment for five weeks attenuates NAFLD progression in ApoE^(−/−)^ mice by promoting autophagy, reducing ER stress, and inhibiting hepatic apoptosis. The authors point out that further research is required in order to delineate the possible dose- and duration-dependent differential effects of empagliflozin on NAFLD development and progression. 

The original articles and reviews published in this Special Issue aim to provide new data and discuss the recent advances in the field of atherosclerosis and atherosclerosis-related diseases. An improved understanding of the common pathophysiological processes that result in cardio-metabolic diseases will help in the identification of effective prevention strategies and the development of innovative therapeutic targets which can be introduced in routine clinical practice.

## References

[1] Meng H., Ruan J., Yan Z., Chen Y., Liu J., Li X., Meng F. (2022). New Progress in Early Diagnosis of Atherosclerosis. Int. J. Mol. Sci..

[2] Kowara M., Cudnoch-Jedrzejewska A. (2021). Different Approaches in Therapy Aiming to Stabilize an Unstable Atherosclerotic Plaque. Int. J. Mol. Sci..

[3] Kassi E., Spilioti E., Nasiri-Ansari N., Adamopoulos C., Moutsatsou P., Papapanagiotou A., Siasos G., Tousoulis D., Papavassiliou A.G. (2015). Vascular Inflammation and Atherosclerosis: The Role of Estrogen Receptors. Curr. Med. Chem..

[4] Nasiri-Ansari N., Spilioti E., Kyrou I., Kalotychou V., Chatzigeorgiou A., Sanoudou D., Dahlman-Wright K., Randeva H.S., Papavassiliou A.G., Moutsatsou P. (2022). Estrogen Receptor Subtypes Elicit a Distinct Gene Expression Profile of Endothelial-Derived Factors Implicated in Atherosclerotic Plaque Vulnerability. Int. J. Mol. Sci..

[5] Dimitropoulos S., Mystakidi V.C., Oikonomou E., Siasos G., Tsigkou V., Athanasiou D., Gouliopoulos N., Bletsa E., Kalampogias A., Charalambous G. (2020). Association of Soluble Suppression of Tumorigenesis-2 (ST2) with Endothelial Function in Patients with Ischemic Heart Failure. Int. J. Mol. Sci..

[6] Chen Y.C., Sheu J.J., Chiang J.Y., Shao P.L., Wu S.C., Sung P.H., Li Y.C., Chen Y.L., Huang T.H., Chen K.H. (2020). Circulatory Rejuvenated EPCs Derived from PAOD Patients Treated by CD34(+) Cells and Hyperbaric Oxygen Therapy Salvaged the Nude Mouse Limb against Critical Ischemia. Int. J. Mol. Sci..

[7] Taberner-Cortes A., Vinue A., Herrero-Cervera A., Aguilar-Ballester M., Real J.T., Burks D.J., Martinez-Hervas S., Gonzalez-Navarro H. (2020). Dapagliflozin Does Not Modulate Atherosclerosis in Mice with Insulin Resistance. Int. J. Mol. Sci..

[8] Dimitriadis G.K., Nasiri-Ansari N., Agrogiannis G., Kostakis I.D., Randeva M.S., Nikiteas N., Patel V.H., Kaltsas G., Papavassiliou A.G., Randeva H.S. (2019). Empagliflozin improves primary haemodynamic parameters and attenuates the development of atherosclerosis in high fat diet fed APOE knockout mice. Mol. Cell. Endocrinol..

[9] Nasiri-Ansari N., Dimitriadis G.K., Agrogiannis G., Perrea D., Kostakis I.D., Kaltsas G., Papavassiliou A.G., Randeva H.S., Kassi E. (2018). Canagliflozin attenuates the progression of atherosclerosis and inflammation process in APOE knockout mice. Cardiovasc. Diabetol..

[10] Sagris M., Vardas E.P., Theofilis P., Antonopoulos A.S., Oikonomou E., Tousoulis D. (2021). Atrial Fibrillation: Pathogenesis, Predisposing Factors, and Genetics. Int. J. Mol. Sci..

[11] Aguilar-Ballester M., Hurtado-Genoves G., Taberner-Cortes A., Herrero-Cervera A., Martinez-Hervas S., Gonzalez-Navarro H. (2021). Therapies for the Treatment of Cardiovascular Disease Associated with Type 2 Diabetes and Dyslipidemia. Int. J. Mol. Sci..

[12] Sun W., Wang Y., Zheng Y., Quan N. (2020). The Emerging Role of Sestrin2 in Cell Metabolism, and Cardiovascular and Age-Related Diseases. Aging Dis..

[13] Kishimoto Y., Kondo K., Momiyama Y. (2021). The Protective Role of Sestrin2 in Atherosclerotic and Cardiac Diseases. Int. J. Mol. Sci..

[14] Matloch Z., Cinkajzlova A., Mraz M., Haluzik M. (2018). The Role of Inflammation in Epicardial Adipose Tissue in Heart Diseases. Curr. Pharm. Des..

[15] Conceicao G., Martins D., Miranda M.I., Leite-Moreira A.F., Vitorino R., Falcao-Pires I. (2020). Unraveling the Role of Epicardial Adipose Tissue in Coronary Artery Disease: Partners in Crime?. Int. J. Mol. Sci..

[16] Eitan A., Roguin A. (2016). Coronary artery ectasia: New insights into pathophysiology, diagnosis, and treatment. Coron. Artery Dis..

[17] Richards G.H.C., Hong K.L., Henein M.Y., Hanratty C., Boles U. (2022). Coronary Artery Ectasia: Review of the Non-Atherosclerotic Molecular and Pathophysiologic Concepts. Int. J. Mol. Sci..

[18] Nasiri-Ansari N., Androutsakos T., Flessa C.M., Kyrou I., Siasos G., Randeva H.S., Kassi E., Papavassiliou A.G. (2022). Endothelial Cell Dysfunction and Nonalcoholic Fatty Liver Disease (NAFLD): A Concise Review. Cells.

[19] Flessa C.M., Kyrou I., Nasiri-Ansari N., Kaltsas G., Kassi E., Randeva H.S. (2022). Endoplasmic reticulum stress in nonalcoholic (metabolic associated) fatty liver disease (NAFLD/MAFLD). J. Cell Biochem..

[20] Flessa C.M., Kyrou I., Nasiri-Ansari N., Kaltsas G., Papavassiliou A.G., Kassi E., Randeva H.S. (2021). Endoplasmic Reticulum Stress and Autophagy in the Pathogenesis of Non-alcoholic Fatty Liver Disease (NAFLD): Current Evidence and Perspectives. Curr. Obes. Rep..

[21] Flessa C.M., Nasiri-Ansari N., Kyrou I., Leca B.M., Lianou M., Chatzigeorgiou A., Kaltsas G., Kassi E., Randeva H.S. (2022). Genetic and Diet-Induced Animal Models for Non-Alcoholic Fatty Liver Disease (NAFLD) Research. Int. J. Mol. Sci..

[22] Androutsakos T., Nasiri-Ansari N., Bakasis A.D., Kyrou I., Efstathopoulos E., Randeva H.S., Kassi E. (2022). SGLT-2 Inhibitors in NAFLD: Expanding Their Role beyond Diabetes and Cardioprotection. Int. J. Mol. Sci..

[23] Nasiri-Ansari N., Nikolopoulou C., Papoutsi K., Kyrou I., Mantzoros C.S., Kyriakopoulos G., Chatzigeorgiou A., Kalotychou V., Randeva M.S., Chatha K. (2021). Empagliflozin Attenuates Non-Alcoholic Fatty Liver Disease (NAFLD) in High Fat Diet Fed ApoE((−/−)) Mice by Activating Autophagy and Reducing ER Stress and Apoptosis. Int. J. Mol. Sci..

